# Neuroimaging and kinematic biomarkers of post-stroke upper limb motor impairment

**DOI:** 10.1016/j.nicl.2025.103854

**Published:** 2025-07-28

**Authors:** Joyce L. Chen, Timothy K. Lam, Melanie C. Baniña, Daniele Piscitelli, Mindy F. Levin

**Affiliations:** a Faculty of Kinesiology and Physical Education, University of Toronto, Toronto, ON M5S 2W6, Canada; b Canadian Partnership for Stroke Recovery, Hurvitz Brain Sciences Research Program, Sunnybrook Research Institute, Toronto, ON M4N 3M5, Canada; c School of Physical and Occupational Therapy, McGill University, Montreal, QC H3G 1Y5, Canada; d Feil/Oberfeld Research Centre of the Jewish Rehabilitation Hospital/Centre for Interdisciplinary Research in Rehabilitation, Laval, QC H7V 1R2, Canada; e Department of Kinesiology, University of Connecticut, Storrs, CT 06269, USA

**Keywords:** Motor, Restitution, Compensation, Upper extremity, Corticospinal tract

## Abstract

Structural and functional biomarkers derived from magnetic resonance imaging explain some variance in post-stroke motor impairment. The understanding of the nature of impairment and the discrimination between true behavioural motor recovery/restitution and motor compensation may be improved by the addition of kinematic information. The aim of the study was to determine the influence of neuroimaging combined with kinematic biomarkers in explaining the variance in motor impairment of the upper limb. People living with late sub-acute to chronic stroke (n = 25) underwent the Fugl Meyer Assessment – Upper Limb (FMA-UL), magnetic resonance imaging, and completed a reaching task where upper limb and trunk kinematics were recorded. Regression analyses were performed to determine the amount of variability in FMA-UL explained by the following biomarkers: the amount of corticospinal tract impacted by the stroke lesion (CST involvement), interhemispheric and ipsilesional resting state connectivity, and the Trunk-based Index of Performance (IPt) that measures skilled reaching ability while accounting for trunk compensation. CST involvement, interhemispheric connectivity, and the IPt, together explained ∼49 % of the variance in the FMA-UL (F(3,21) = 8.694, *p* = 0.001, R^2^_adj_ = 0.49). The IPt explained an additional 14 % of the variance in the FMA-UL compared to CST involvement alone (p = 0.02). The IPt is a relevant kinematic biomarker of post-stroke upper limb motor impairment. Our findings suggest the importance of using multiple categories of biomarkers to better understand the level of post-stroke motor impairment.

## Highlights

• Biomarkers provide insight on how movements are affected in people living with stroke.
• Neuroimaging and kinematic biomarkers explain 49% variance in motor impairment.
• The amount of corticospinal tract affected by stroke accounts for 27% variance in motor impairment.
• A kinematic biomarker of skilled reaching accounts for 14% variance in motor impairment.
• The resting state motor connectivity accounts for 8% variance in motor impairment.

## 1 Introduction

An important priority in stroke rehabilitation research is the identification of biomarkers that predict post-stroke motor outcomes and the potential for change (i.e., improvement) in motor behaviour (Boyd et al., 2017, Stinear, 2017). A biomarker is an indicator of a biological or pathological process, or a response to an intervention (Califf, 2018). Biomarkers derived from neuroimaging and/or non-invasive brain stimulation approaches may provide insight on a person’s neurobiological potential for true motor recovery/restitution (Stinear, 2017). In this paper, the term recovery is used in two contexts. First, when used in the context of ‘recovery’, the term refers to improvements in motor behaviour over time. Second, when used in the context of “recovery/restitution”, the term refers to the ability to perform movement (e.g. spatial/temporal interjoint coordination) in the same manner as it was performed before the stroke (Levin et al., 2009). Several candidate neurobiological biomarkers related to motor recovery have been identified (Boyd et al., 2017, Kim and Winstein, 2017) with some validated against clinical measures of upper limb impairment (Smith et al., 2019, Stinear et al., 2017). However, there is growing interest in identifying kinematic variables as biomarkers of motor impairment that reflect the quality of movement patterns at the underlying motor impairment level (Scott et al., 2023, Kwakkel et al., 2019), and thus can differentiate between true motor recovery/restitution and behavioural compensation (Levin et al., 2009, Kwakkel et al., 2019, Ward, 2023). Therefore, we aimed to better understand whether and which biomarkers at the neuroimaging and kinematic levels contribute to post-stroke clinical motor impairment as measured with the Fugl-Meyer Assessment of the Upper Limb (FMA-UL) (Fugl-Meyer et al., 1975). The FMA-UL is a commonly used clinical and research assessment, but is not without limitations (Scott et al., 2023, Lohse et al., 2021, Gladstone et al., 2002, Bonkhoff et al., 2020, See et al., 2013). Specifically, we also asked whether the addition of a kinematic biomarker of movement quality improves the amount of variance explained in the FMA-UL.

Neuroimaging biomarkers assay the brain’s structure and function and thus, the neurobiological effects of stroke (Boyd et al., 2017, Stinear, 2017). Structural biomarkers delineate how the stroke affects white matter pathways such as the corticospinal tract (CST). The amount of CST affected by the stroke correlates with clinical motor impairment and predicts motor recovery (Lin et al., 2019, Feng et al., 2015). The CST biomarker explains between 20 and 80 % of the variance in upper limb motor impairment (Quinlan et al., 2015, Lam et al., 2018, Pineiro et al., 2000, Zhu et al., 2010) and is recommended for use in clinical and research contexts (Boyd et al., 2017). The range in explained variance may be attributed to different methodologies used to delineate the CST biomarker, and as well, the outcomes used to represent motor impairment. We recently compared four different approaches that quantify the structural integrity of the CST using anatomical magnetic resonance imaging (MRI) scans (Schuch et al., 2022). We showed that the transverse MRI slice with the maximum percentage of overlap between the stroke lesion and CST (herein referred to as “CST involvement”) accounted for the most variance (∼30 %) in the FMA-UL in people with chronic stroke (Schuch et al., 2022). Thus, this approach has been adopted in the present study.

Functional neuroimaging biomarkers reflect how the stroke affects grey matter regions and their connected networks, and are commonly delineated using connectivity metrics (Grefkes and Fink, 2014). Resting state fMRI (rsfMRI) connectivity is of interest in stroke research because data are obtained without the need of participants performing a task, removing the confound of motor performance on neural activation (Carter et al., 2012). rsfMRI connectivity between homologous ipsilesional and contralesional sensorimotor regions, as well as connectivity between other networks, relate to post-stroke motor outcomes and recovery (Carter et al., 2010, Paul et al., 2021, Ovadia-Caro et al., 2013, Ismail et al., 2024), though see other work (Branscheidt et al., 2022, Nijboer et al., 2017). A recent systematic review found interhemispheric rsfMRI connectivity between left and right primary motor cortex (M1-M1 connectivity) to be commonly associated with motor recovery (Ismail et al., 2024). It is thought that there may be two distinct processes associated with neuronal reorganization in the motor system post-stroke (Paul et al., 2021, Paul et al., 2023). The first, interhemispheric reorganization, may support influences of bilateral motor regions onto ipsilesional M1, and/or offer an alternative pathway via contralesional M1 for motor commands (Paul et al., 2023). The second, ipsilesional reorganization, may reflect task-specific compensation within the ipsilesional hemisphere to support task performance (Paul et al., 2021). Therefore, we sought to evaluate the associations of both interhemispheric M1-M1 and intra-hemispheric motor rsfMRI connectivity to motor impairment.

Kinematic measures more precisely describe how specific tasks are performed (Scott et al., 2023, Subramanian et al., 2010), and thus could represent a class of biomarkers. Kinematics can be characterized at the motor performance level (i.e., endpoint accuracy, speed, trajectory smoothness and straightness) and at the movement quality level (i.e., spatial–temporal characteristics of individual joint movements and inter-joint coordination) (Levin et al., 2009). Detailed kinematics at these two levels can be used to distinguish between whether improvements in task performance are due to true motor recovery/restitution or compensation. Previously, specific kinematic descriptors have been suggested as potential biomarkers for motor impairment but these have all been related to well-defined ‘prescribed’ motor actions such as drinking from a cup (Alt Murphy et al., 2018) or reaching for a target where little variance is expected (Subramanian et al., 2010, Hussain et al., 2019). We recently developed a new metric based on Fitts’ Law. Fitts’ Law is a measure of fundamental motor skill that quantifies skilled performance in a single measure. It states that the time to reach a target is based on the distance moved and the size of the target (Fitts, 1954). Our new measure, Trunk-based Index of Performance (IPt) (Piscitelli et al., 2023), is more specific for measuring performance in patient populations since it incorporates both upper limb motor performance (endpoint accuracy and speed) and movement quality (trunk displacement [compensation]) elements. Thus, the IPt incorporates various kinematic variables into a single measure and reflects skilled performance. We propose that it may be a relevant kinematic biomarker that distinguishes between motor impairment associated with true motor recovery/restitution and compensation.

The IPt measures performance of an ecological task at a fundamental motor skill level and thus may contribute information about upper limb motor impairment as measured with the FMA-UL. The FMA-UL is a clinical assessment that evaluates motor impairment and is thought to quantify the amount of behavioural recovery/restitution of movements, if scored properly (See et al., 2013). However, indirect evidence suggests that the FMA-UL may not fully capture restitution (Levin et al., 2023). A single proof-of-concept study found that scores on the FMA improved after an upper limb intervention, without improvement in upper limb kinematics (Levin et al., 2023). This may suggest that improvements in the FMA-UL were due to increased use of compensations. An important consideration is that the FMA-UL uses an ordinal criteria-based scale to evaluate the extent to which a movement can be performed (0 – cannot perform, 1 – performs partially, 2 – performs fully). In contrast, the IPt provides more granular information about how movements are performed, which may yield information about compensation not captured by the FMA-UL. Taken together, obtaining information about how kinematics influence the FMA-UL score is important. Therefore we hypothesize that the IPt, which provides a measure of the degree of restitution/compensation while reaching, explains some impairment component of the FMA-UL. Further, we hypothesize that the amount of CST involvement, M1-M1 connectivity, ipsilesional connectivity and the IPt would explain the most variability in the FMA-UL than any biomarker alone.

## 2 Materials and Methods

### 2.1 Participants

Twenty-five participants with late sub-acute to chronic stroke provided informed written consent to participate in a two-site double-blind, crossover randomized controlled trial (https://www.clinicaltrials.gov, NCT02473549). The study was approved by the Research Ethics Boards at Sunnybrook Health Sciences Centre (Toronto) and the Centre for Interdisciplinary Research in Rehabilitation (Montreal). In this paper, we analyzed and report a subset of the MRI and baseline data from the randomized controlled trial.

Participants were recruited via referrals from tertiary rehabilitation hospitals in Canada (Toronto, Ontario, and Montreal and Laval, Quebec). Inclusion criteria were: unilateral first-time ischemic stroke in the middle cerebral artery territory, stroke onset >3 months and <5 years (to minimize potential influence of co-morbities), age >18 years, mild to severe motor deficits as defined by a Chedoke-McMaster Stroke Assessment Impairment Inventory Stage of Arm (CMSA-Arm) ≥3) (Gowland et al., 1995), the ability to perform approximately 30° of active elbow extension, and English or French speaking. Exclusion criteria were: significant cognitive deficit (<23/30, Montreal Cognitive Assessment) (Dong et al., 2010), severe apraxia (2 standard deviations below the mean of a healthy control sample, Waterloo Apraxia test) (Stamenova et al., 2010), neglect (>40/100, Sunnybrook Neglect Assessment Procedure) (Leibovitch et al., 2012), history of musculoskeletal problems in the upper limb and/or back, neurodegenerative or psychiatric disease, contraindications to MRI, and/or taking medications known to modulate the effects of transcranial direct current stimulation (Cohen-Kadosh, 2014) (applied in main clinical trial).

### 2.2 Motor assessments

Motor impairment was assessed with the FMA-UL (Gladstone et al., 2002) that evaluates reflex activity, voluntary movements, and upper limb coordination. Each task on the FMA-UL is scored using a 3-point scale from 0 (cannot be performed) to 2 points (fully performed) with the total score ranging from 0 (severe impairment) to 66 points (normal movement). Aside from the total score, we also considered an FMA-UL arm sub-score that describes only the movements of the proximal arm in Section A of the scale, for a total of 36 points. All clinical assessments were conducted by licensed health care professionals (one occupational therapist and two physical therapists).

### 2.3 Reaching test task

**Fig. 1:**
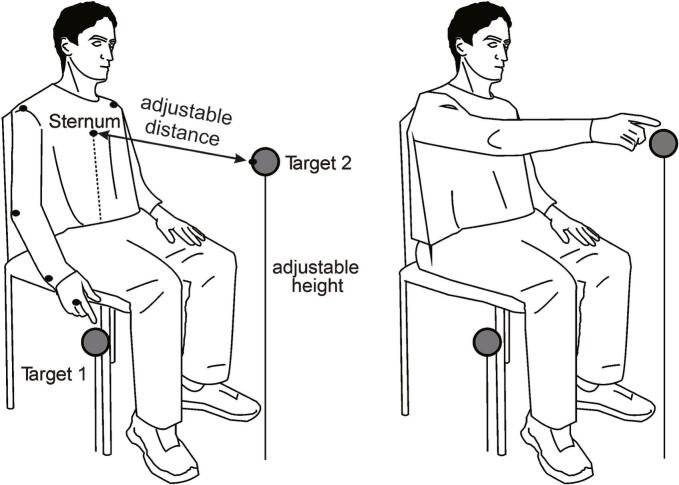
Set up for the Reaching Test Task. Black dots represent motion capture infrared emitting diodes.

Functional ability was assessed using a standardized reaching test task (Cirstea and Levin, 2000). Participants were seated in an armless chair with their back supported by a backrest. In the initial position, the shoulder was in 0° flexion, the elbow was in 40° flexion, the wrist was in 20° extension, the hand/forearm was in the neutral supination/pronation position, and the hand rested on a platform (Fig. 1).

Participants were instructed to reach-to-touch a target using their third metacarpal (hand in a closed fist) with their eyes opened, and then to return the hand to the starting position. This constituted one trial and was repeated for a total of 25 trials. A closed fist was used to accommodate participants without sufficient finger movement such that performance could be compared across all participants. The target was set at the trunk midline and at the height of the sternal notch. The distance of the target was set at arm length, measured from the medial mid-axillary border to the tip of the third metacarpal, with full elbow extension. Participants performed five practice trials to familiarize themselves with the procedure prior to recording.

### 2.4 Kinematic data acquisition and analysis

Upper limb and trunk kinematics were recorded on an Optotrak Certus motion capture system (Northern Digital, Inc., Waterloo, Canada). Seven markers (i.e., infrared-emitting diodes) were used to acquire the kinematic data at a sampling rate of 100 Hz. Markers were placed on the ipsilateral endpoint (third metacarpal head), wrist (ulnar head), elbow (lateral epicondyle), both shoulders (ipsilateral and contralateral acromion processes), trunk (mid-sternum), and the target (Fig. 1).

For each test task trial, movement onset and offset were determined from the tangential velocity trace of the endpoint marker as the times when the velocity trace rose (or fell) and remained above (or below) 10 % of the mean peak velocity of the trial for a minimum of 25 ms. Three kinematic measures were extracted from each trial and used to calculate the IPt (Piscitelli et al., 2023) (described below): 1) trajectory length (*D*, in mm): sagittal distance moved by the endpoint marker (y coordinate); 2); movement time (*MT*, in ms): duration of the reach from movement onset to offset; and 3) trunk displacement (*t*, in mm): sagittal distance moved by the sternal marker from movement onset to offset. The initial vertical position of the trunk was defined as 0 mm of trunk displacement (i.e., resting trunk origin). Data preprocessing involved interpolation using a third-order spline function and low-pass filtering at 20 Hz. All kinematic data were analyzed using custom software in MATLAB R2018a (MathWorks, Natick, MA).

### 2.5 Trunk-based Index of performance (IPt)

The Trunk-based Index of Performance (IPt) is a measure that has its theoretical foundation in Fitts law. Fitts (1954) proposed an Index of Performance (IP) as a measure of human performance (Fitts, 1954). This is often referred to as the speed-accuracy trade-off relationship. The IP is defined as:

$$IP=\frac{\mathrm{ID}}{\mathrm{MT}}$$

where *MT* is the movement time and *ID* is the index of difficulty.

Fitts’ Law states that the time required to move the endpoint to a target (*MT*) depends on the distance to the target (*D*) and the target width (*W*) (Fitts, 1954). This relationship is described by the following equation (Guiard and Olafsdottir, 2011, MacKenzie, 1992):

$$\mathrm{MT}=a+b\left[{log}_{2}\left(\frac{2D}{W}+1\right)\right]$$

Reaching a target that is closer (i.e., smaller D) and larger (i.e., larger W) is easier and results in a shorter movement time. Conversely, reaching a farther (i.e., larger D) and smaller (i.e., smaller W) target is more difficult and requires a longer movement time.

For a given target acquisition task, the Index of Difficulty (*ID)* quantifies task difficulty based on various combinations of *D* and *W*, is measured in ‘bits’, and defined by:

$${log}_{2}\left(\frac{2D}{W}+1\right)$$

Taken together, the IP therefore combines speed (e.g. movement time) and accuracy components of a target acquisition task in a single measure.

Recently, we used the Index of Difficulty to quantify the Index of Performance in a reaching task in people with stroke (Piscitelli et al., 2023). However, to account for typical trunk compensatory movements during upper limb reaching tasks (Cirstea and Levin, 2000, Michaelsen and Levin, 2004), we modified the equation as follows:

$$\mathrm{IDe}=\log_{2}\left(\frac{\frac{\sum_{i=1}^{N}\left(D-t\right)}{N}}{\mathrm{We}}+1\right)$$

where *t* is the forward trunk displacement in the sagittal plane and *N* is the number of trials. *We* is the effective target width computed as the standard deviation of the total error of all trials multiplied by the 95 % CI z-value, i.e., *We* = 1.96*SD_TotalError. Total error is the distance of the endpoint marker from the target position. The effective target width, *We*, accounts for endpoint spatial variability (Fitts and Peterson, 1964, MacKenzie, 2018). Therefore, we coined the Index of Difficulty in our study as the Effective Index of Difficulty (*IDe)* because our metric uses the effective target width. Similarly, we coined the Index of Performance in our study as Index of Performance-trunk (*IPt*) because our metric incorporates trunk displacement.

The IPt was calculated for each test task trial and averaged across all trials for each participant to obtain a single IPt value representative of reaching performance in the hemiparetic limb. A larger IPt value signifies better motor skill performance.

### 2.6 Magnetic resonance imaging (MRI) acquisition

Magnetic resonance images were acquired on a Siemens Magnetom Prisma 3 Tesla system (Erlangen, Germany). MRI scans were obtained either at the Sunnybrook Health Sciences Centre (Toronto) or the Montreal Neurological Institute (Montreal), with identical MRI parameters. We acquired T1-weighted high resolution anatomical, fluid-attenuated inversion recovery (FLAIR), and rsfMRI scans. Scan parameters were: T1-weighted: TR = 2300 ms; TE = 2.05 ms; flip angle = 9°; voxel size = 1 × 1 × 1 mm^3^; field of view (FOV) = 256 × 256 mm^2^; matrix size = 256 × 256 voxels; 192 axial slices; slice thickness = 1 mm. FLAIR: TR = 9000 ms; TE = 119 ms; flip angle = 165°; voxel size = 0.9 × 0.9 × 3 mm^3^; FOV = 240 × 240 mm^2^; matrix size = 256 × 256 voxels; 48 axial slices; slice thickness = 3 mm. Rs-fMRI: TR = 2400 ms; TE = 30 ms; flip angle = 70°; voxel size = 3.5 × 3.5 × 3.5 mm^3^; FOV = 224 × 224 mm^2^; matrix size = 64 × 64 voxels; 41 axial slices; slice thickness = 3.5 mm. The scan duration was 10.21 min with 255 volumes. Participants were instructed to fixate on a cross displayed during the rsfMRI scan. Physiological information was also recorded during the rs-fMRI scan using a pulse oximeter placed on the participant’s finger and a respiration belt around the chest to measure heart beat and respiration, respectively.

### 2.7 Stroke lesion

Lesions were manually traced using the imaging software FMRIB Software Library (FSL) (Jenkinson et al., 2012) version 5.0.2. A trained researcher (TKL), blinded to participant’s demographics and FMA-UL score, manually traced the stroke lesion on T1-weighted anatomical scans. FLAIR images were also reviewed to confirm the lesion tracings. These tracings were used to create a lesion mask for each participant. All lesion masks were subsequently reviewed by an experienced research radiologist to verify the tracing accuracy.

Lesion masks for each participant were registered to the Montreal Neurological Institute – International Consortium of Brain Mapping (MNI-ICBM) 152 non-linear (2 mm) space using an affine transformation and FMRIB’s Non-Linear Image Registration Tool (FNIRT) in FSL (Jenkinson et al., 2012). We used the -inweight option whereby voxels in the lesion mask were denoted with 0 and voxels outside were denoted with 1. Thus, voxels in the lesion were excluded during the registration procedure.

### 2.8 Corticospinal tract (CST) involvement calculation

**Fig. 2:**
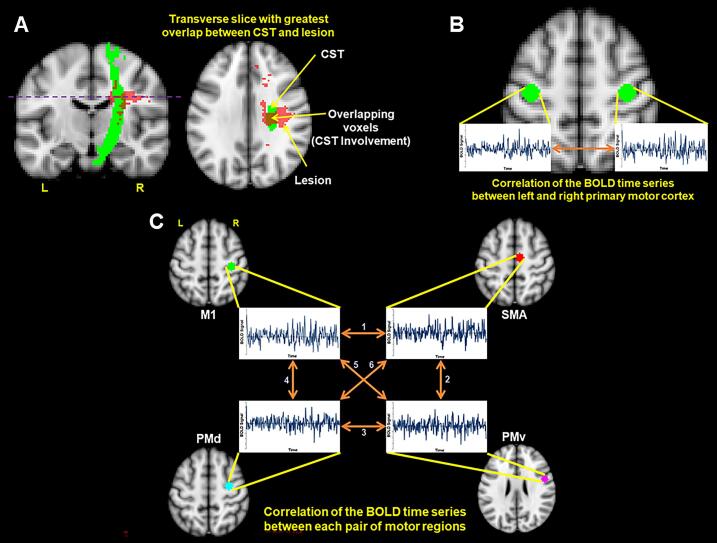
Neuroimaging-derived biomarkers. (**A**) Schematic of the corticospinal tract (CST) involvement calculation. The purple dotted line represents the transverse slice of the CST template (green) with the greatest overlap with the lesion (red). This transverse slice was used to calculate CST involvement. (**B**) Schematic of the left and right primary motor cortex (M1-M1) connectivity. The BOLD time series from the left and right M1 seeds were extracted from the resting state functional magnetic resonance imaging (rs-fMRI) data. A Pearson’s correlation was computed between the pair of BOLD time series and a Fisher’s r-to-z transformation was applied for a measure of M1-M1 connectivity (**C**) Schematic of the ipsilesional connectivity. The BOLD time series from the right M1, right supplementary motor area (SMA), right dorsal premotor cortex (PMd), and right ventral premotor cortex (PMv) were extracted from the rs-fMRI data. Pearson’s correlations were computed between each pair of time series: 1) M1-SMA; 2) SMA-PMv; 3) PMd-PMv; 4) M1-PMd; 5) M1-PMv; and 6) SMA-PMd. A Fisher’s r-to-z transformation was applied to all six r-values and the average was computed for a composite measure of ipsilesional connectivity. For consistency across each biomarker calculation, the lesion masks and rs-fMRI images were flipped along the mid-sagittal plane (if necessary) such that all lesions are displayed on the right hemisphere. Abbreviations: “L” indicates left; “R” indicates right. (For interpretation of the references to colour in this figure legend, the reader is referred to the web version of this article.)

CST involvement was defined as the amount of overlap between the stroke lesion of a participant and a normative CST template, represented as a percentage. Lesion masks registered in standard space were used for the calculation of the amount of CST involvement. If required, lesion masks were flipped along the mid-sagittal plane such that all lesions were displayed on the right hemisphere. This allowed for the same right hemisphere CST template to be used in the CST involvement calculation across all participants. The CST template was obtained from the Johns Hopkins University (JHU) white matter tractography atlas (Hua et al., 2008) in FSL. A binary mask of the right primary motor cortex (M1) CST, with no threshold applied, was used as the CST template. Our approach for delineating the amount of CST involvement is based on the method first described by Pineiro et al. (Pineiro et al., 2000) and applied in our previous work (Lam et al., 2018, Schuch et al., 2022, Lam et al., 2020). We determined the transverse slice of the CST with the greatest overlap with the stroke lesion (Fig. 2A) and applied the following equation:

$$\begin{array}{c} \mathrm{CST}\;\mathrm{Involvement}\\ \;=\frac{\mathrm{Number}\;\mathrm{of}\;\mathrm{overlapping}\;\mathrm{voxels}\;\mathrm{between}\;\mathrm{the}\;\mathrm{CST}\;\mathrm{and}\;\mathrm{lesion}\;\mathrm{for}\;\mathrm{the}\;\mathrm{transverse}\;\mathrm{slice}}{\mathrm{Total}\;\mathrm{numbe}r\;\mathrm{of}\;\mathrm{CST}\;\mathrm{voxels}\;\mathrm{for}\;\mathrm{the}\;\mathrm{transverse}\;\mathrm{slice}}\\ \;\times 100\% \end{array}$$

The CST involvement values range from 0 % (no overlap between CST template and lesion mask across all transverse slices of the CST template) to 100 % (full overlap between CST template and lesion mask for at least one transverse slice of the CST template).

### 2.9 Resting state functional magnetic resonance imaging (rsfMRI)

#### 2.9.1 Preprocessing

Physiological data were regressed out from the rsfMRI images using the RETROICOR algorithm (Glover et al., 2000) in AFNI (Cox, 1996). The remaining data preprocessing and analyses were conducted using FSL version 5.0.2 (Jenkinson et al., 2012). If required, the rsfMRI images were flipped along the mid-sagittal plane such that the stroke lesion was in the right hemisphere. This enabled the ipsilesional (i.e., right) hemisphere to be uniform across participants that allowed the motor connectivity calculation (described below) to be conducted with the same motor seeds for all participants. We also did not exclude lesion voxels from the seed masks to ensure that the number of voxels in the seed masks for each participant remained constant.

The FMRI Expert Analysis Tool (FEAT) was used to preprocess the rsfMRI images, which included the following steps: head motion correction, slice time correction to the middle slice, spatial smoothing with a Gaussian kernel of 6 mm full width at half maximum, and high pass temporal filtering at 0.01 Hz. Non-brain tissue (i.e., skull) was also removed using the Brain Extraction Tool (BET) in FSL.

Nuisance regressors pertaining to head motion, cerebrospinal fluid (CSF), and white matter (WM) were extracted from the rsfMRI data and inputted as regressors of no interest in the General Linear Model (GLM) (see Supplementary Methods). This produced the residual rsfMRI data, free of nuisance variables, from which we extracted the time series in the motor regions of interest to determine the motor resting state connectivity.

#### 2.9.2 Motor connectivity

We applied a seed-based region of interest approach to analyze the rsfMRI connectivity data (Carter et al., 2010, Biswal et al., 1995, Cole et al., 2010). The regions of interest were primary motor cortex (M1), the supplementary motor area (SMA), and the dorsal and ventral premotor cortex (PMd, PMv, respectively). This allowed us to investigate left M1 to right M1 connectivity (M1-M1 connectivity), and connectivity between all motor seeds in the ipsilesional hemisphere (ipsilesional connectivity). To derive the seed coordinates for each region of interest, we first identified all studies from three *meta*-analyses (Mayka et al., 2006, Hardwick et al., 2018, Papitto et al., 2020) that utilized an arm/elbow fMRI or positron emission tomography (PET) paradigm. We extracted the peak arm and/or elbow coordinates from these studies (Mayka et al., 2006, Hardwick et al., 2018, Papitto et al., 2020) and computed the average of each coordinate (see Supplementary Methods). We focused on studies that localized the arm and elbow as this was most related to the reaching test task that also required arm/elbow as opposed to hand/finger movements. The average peak coordinates for the motor regions of interest in standard MNI-ICBM 152 space were: left M1 (−34, −23, 54]; right M1 (34, −23, 54); right SMA (8, −11, 54); right PMd (30, −9, 55); and right PMv (51, 3, 26) (Supplementary Tables S1–S4). A 6 mm radius around each coordinate was then applied to create the M1, SMA, PMd, and PMv seed masks. Seed masks were non-linearly registered to functional space using FNIRT. For the left M1 seed mask, the mean time series of all voxels within the mask was extracted from the residual rsfMRI data. The process was then repeated for the right M1, SMA, PMd, and PMv seed masks. Thus, each participant had five motor time series, one each for the left M1, right M1, SMA, PMd, and PMv seed masks.

To derive the M1-M1 connectivity, Pearson’s correlation was computed between the left M1 and right M1 time series (Fig. 2B). A Fisher’s r-to-z transformation was applied to the r-value for each participant to yield M1-M1 connectivity values with a normal distribution. The z-score for each participant was used in the regression analyses (see *Statistical analyses)*. A larger z-score reflects greater connectivity between left and right M1.

To derive the ipsilesional connectivity, Pearson’s correlations were computed for each pair of time series (i.e., M1-SMA, M1-PMd, M1-PMv, SMA-PMd, SMA-PMv, and PMd-PMv), for a total of six r-values (Fig. 2C). A Fisher’s r-to-z transformation was applied to the r-values to yield connectivity values with a normal distribution. Therefore, each participant had six z-scores, each corresponding to the six pairs of time series listed above. To derive a composite value for ipsilesional connectivity, the average of the six z-scores was computed for each participant. The average z-score for ipsilesional connectivity was used for the regression analyses. A larger z-score reflects greater connectivity between the ipsilesional motor regions than a smaller z-score.

### 2.10 Statistical analyses

Regression analyses were performed to determine the amount of variability in the FMA-UL score (dependent variable) explained by each biomarker (i.e., IPt, CST involvement, M1-M1 connectivity, and ipsilesional connectivity). We also performed the same analyses using the FMA-UL arm sub-score, details of which are reported in the Supplemental Results. First, a simple linear regression model was computed whereby the IPt was included as the explanatory variable to determine the explained variance in the FMA-UL score. This process was then repeated for each variable. This led to a total of four univariate linear regression models.

Next, a stepwise regression analysis was conducted to determine whether the combined influence of neuroimaging and kinematic biomarkers improved the unique variance explained in the FMA-UL score. The stepwise approach is data-driven as explanatory variables are selected with an F-statistic equal to *p* < 0.05 to be included in the model and those with an F-statistic with *p* > 0.10 to be excluded. The R^2^, adjusted R^2^, and change in adjusted R^2^ (ΔR^2^_adj_) are reported for each model. The beta (β)-values, 95 % confidence intervals, and associated *p*-value for each term in the regression models are also reported. The residuals from the final model of the stepwise regression analysis were normally distributed according to the Shapiro-Wilk statistic. Collinearity between the explanatory variables in the regression model was also evaluated using the variance inflation factor (VIF) and reported in Supplementary Tables S5. VIF values of 10 or greater indicate high multicollinearity (Thompson et al., 2017). For each of our stepwise models, the VIF values for the explanatory variables were small, (i.e. range: 1.00–1.08), which suggests that multicollinearity was not an issue in our models.

Given that the measure of ipsilesional connectivity is a composite of six connectivity metrics, we also performed a Lasso regression whereby we included each of the six connectivity metrics, along with the original explanatory variables. The Lasso regression is appropriate to use when there is a large number of explanatory variables compared to the number of observations. This approach allowed us to identify the most important features in the dataset. Thus, there were a total of 10 explanatory variables that were entered into the Lasso Regression Model: a) the original explanatory variables we tested and reported in the stepwise regression model: CST involvement, M1-M1 connectivity, ipsilesional connectivity, IPt; b) the additional explanatory variables that break down ipsilesional connectivity between different right hemisphere seeds into its constituent components: PMd – PMv; M1 – SMA; M1 – PMv; M1 – PMd; SMA – PMd; SMA – PMv.

In the Supplementary Results, we also report analyses that account for the overlap between the lesion and seed mask, which occurred in n = 5 participants. In Supplementary Tables S6, we also report Spearman corrrelations between demographic variables (age, sex, time since stroke, lesioned hemisphere, and dominant hand affected) and each explanatory variable. Side of lesion and dominant hand affected were correlated with CST involvement. However, including these variables into the stepwise regression model did not change the overall result; they did not significantly explain the FMA-UL or influence the relationship between the biomarkers and FMA-UL. All statistics were performed with SPSS version 22.0 (IBM Corp., Armonk, NY, USA). The exception was the Lasso regression, which was performed in SAS (Statistical Analysis System) software.

## 3 Results

**Fig. 3:**
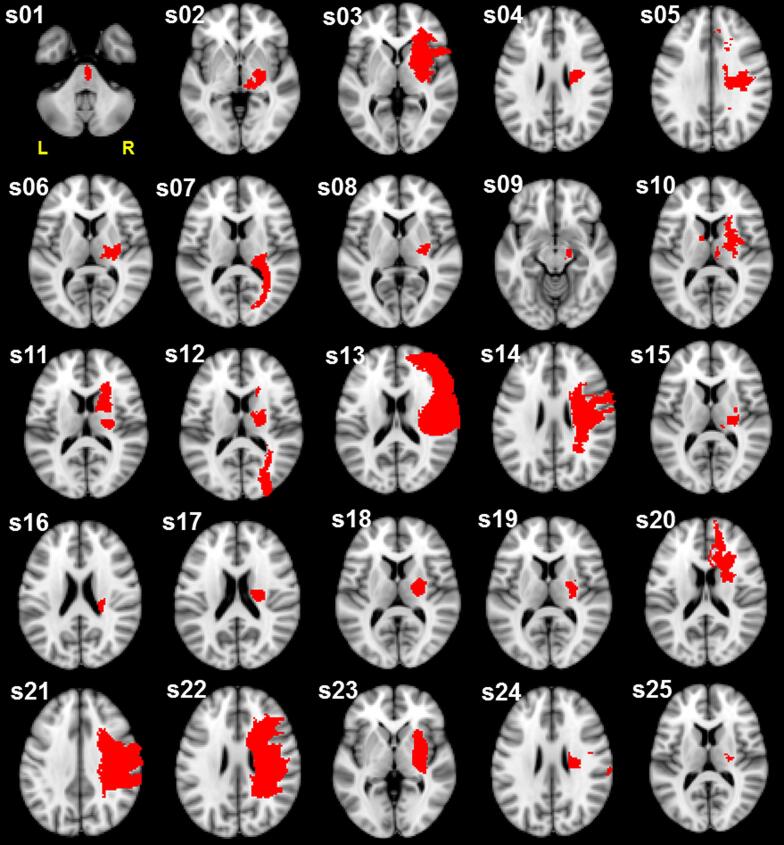
Stroke lesion tracings. Lesion masks (red) were flipped along the mid-sagittal plane (if necessary) for display on the right hemisphere and overlaid on a Montreal Neurological Institute – International Consortium of Brain Mapping (MNI-ICBM) 152 non-linear (2 mm) brain template. The transverse slice of the lesion with the largest cross-sectional area is displayed for each participant. Abbreviations: “s” indicates subject; “L” indicates left; “R” indicates right. (For interpretation of the references to colour in this figure legend, the reader is referred to the web version of this article.)

Demographic, motor performance, and biomarker data for the twenty-five participants are summarized in Table 1 (group data) and Supplementary Tables S7 and S8 (individual data). Lesion tracings for each participant are depicted in Fig. 3. Participants had a mix of cortical and subcortical lesions extending into white matter (details are reported in (Schuch et al., 2022).

**Table 1:** Participant demographics.

**Participants**	25
**Demographics**	
Age, years	60.6 ± 11.7 (38 – 86)
Sex	
Male	22 (88%)
Female	3 (12%)
Time since stroke, months	18.4 ± 16.9 (3.6 – 61.3)
Lesioned hemisphere	
Left	12 (48%)
Right	13 (52%)
Lesion volume, cc	24.0 ± 39.7 (0.8 – 193.9)
Dominant hand affected	
Yes	10 (40%)
No	15 (60%)
**Motor assessment**	
Fugl-Meyer Assessment – Upper Limb Score	44.7 ± 12.9 (13 – 65)
**Biomarkers**	
Trunk-based index of performance, bits per second	4.6 ± 2.5 (1.6 – 12.9)
CST involvement, %	61.7 ± 24.9 (13.8 – 100)
M1-M1 connectivity, z-score	0.46 ± 0.20 (0.02 – 0.8)
Ipsilesional connectivity, z-score	0.35 ± 0.14 (0.08 – 0.6)

Data are presented as mean ± standard deviation (range) for continuous variables and count (percentage of sample) for categorical variables.

### 3.1 Simple regression models for the explained variance in FMA-UL

**Fig. 4:**
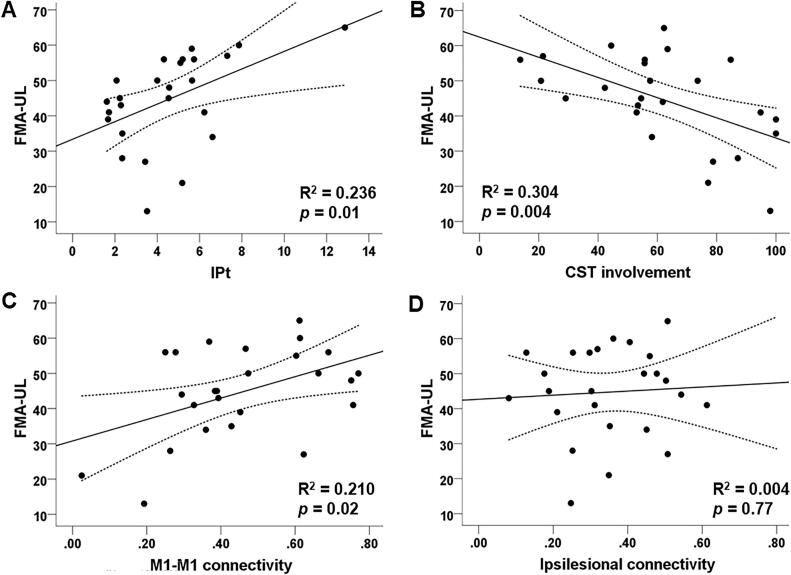
Scatterplots depicting relationships between biomarkers of interest and the Fugl-Meyer Assessment Upper Limb (FMA-UL) scores. The biomarkers included: **A**) trunk-based index of performance (bit/s), **B**) corticospinal tract (CST) involvement (percentage), **C**) left and right primary motor cortex (M1-M1) connectivity (z-score), and **D**) ipsilesional connectivity (z-scores). Dotted lines represent 95% confidence intervals.

The IPt explained 23.6 % of the variance in FMA-UL score (F(1,23) = 7.124, *p* = 0.01). CST involvement explained 30.4 % of the variance in FMA-UL score (F(1,23) = 10.067, *p* = 0.004). M1-M1 and ipsilesional connectivity each explained 21.0 % (F(1,23) = 6.123, *p* = 0.02) and 0.4 % (F(1,23) = 0.09, *p* = 0.77) respectively, as shown in scatterplots in Fig. 4.

### 3.2 Stepwise regression model for the explained variance in FMA-UL

Table 2 summarizes the interim and final models derived from the stepwise regression analysis. From the pool of explanatory variables (IPt, CST involvement, M1-M1 connectivity, ipsilesional connectivity), CST involvement explained the most variance in the FMA-UL score relative to the other explanatory variables, and hence was the first biomarker selected by the statistical software to enter the model (Model 1: F(1,23) = 10.067, *p* = 0.004, R^2^_adj_ = 0.27). The next explanatory variable added into the model was IPt, (Model 2: F(2,22) = 9.335, *p* = 0.001, R^2^_adj_ = 0.41) which explained an additional 14 % of the variance in the FMA-UL score compared to the first model with only CST involvement (*p* = 0.02). The next (and final) explanatory variable added into the model was M1-M1 connectivity (Model 3: F(3,21) = 8.694, *p* = 0.001, R^2^_adj_ = 0.49), which explained an additional 8 % of variance in the FMA-UL score compared to the second model with only CST involvement and IPt (*p* = 0.04). The residuals from the final model were normally distributed. Ipsilesional connectivity was excluded from the final model as its inclusion did not significantly increase the explained variance in the FMA-UL score (β =  − 0.26, *p* = 0.14).

**Table 2:** Stepwise regression for the Fugl-Meyer Assessment Upper Limb (FMA-UL).

**Model**	**R^2^**	**Adjusted R^2^**	***p*-value**	**β[95% CI]**	***p*-value**	**ΔR^2^_adj_**	***p*-value**
Model 1:	0.30	0.27	0.004			–	–
CST involvement				−0.55 [−0.47, −0.1]	0.004		

Model 2:	0.46	0.41	0.001			0.14	0.02
CST involvement				−0.48 [−0.42, −0.08]	0.006		
IPt				0.40 [0.35, 3.74]	0.02		

Model 3:	0.55	0.49	0.001			0.08	0.04
CST involvement				−0.44 [−0.39, −0.07]	0.007		
IPt				0.34 [0.1, 3.32]	0.04		
M1-M1 connectivity				0.32 [0.34, 41.86]	0.04		

A stepwise regression analysis was conducted to determine whether the inclusion of both kinematic and neuroimaging variables increased the explained variance in FMA-UL score. The pool of explanatory variables included the trunk-based index of performance (IPt), corticospinal tract (CST) involvement, left and right primary motor cortex (M1-M1) connectivity, and ipsilesional connectivity. For each model, the R^2^, adjusted R^2^, ΔR^2^, and associated *p*-values are reported. For each term, the beta (β)-values, 95% confidence intervals (CI), and associated *p*-values are reported. The ΔR^2^ for model 2 represents the difference in the adjusted R^2^ between models 1 and 2. The ΔR^2^ for model 3 represents the difference in the adjusted R^2^ between models 2 and 3.

We also performed two additional stepwise regression analyses (see Supplemental Results). For the first analysis, we excluded data from any connectivity pair where one or more seed masks overlapped with the lesion mask. The pattern of findings were similar to those reported above. For the second analysis, we used the FMA-UL arm sub-score as the dependent variable. The model that included the IPt and CST involvement explained approximately 30 % of the variance in the FMA-UL arm sub-score. The amount of variance accounted for by the IPt are similar, 14 % for FMA full score and 18 % for FMA arm sub-score. M1-M1 connectivity was no longer significant in explaining variance in the FMA arm sub-score.

### 3.3 Lasso regression model

The selected model was based on the Schwarz’s Bayesian Criterion (SBC), also known as the Bayesian Information Criterion. The model with the minimal SBC value was chosen. That is, the SBC approach started with the null model (no predictors), then looked for the variable that causes the largest drop in SBC, and so on and so forth until the SBC cannot be reduced further. The model that was significant included the following explanatory variables, in order of amount of explained variance: CST Involvement, IPt, M1-M1 connectivity, and PMd-PMv connectivity (Table 3). Ipsilesional connectivity quantified as a composite average of connectivity values across seed pairs, did not contribute to explaining variance in the FMA-UL in our dataset. Taken together, the results of the Lasso regression converge with the stepwise regression model.

**Table 3:** Lasso regression for the Fugl-Meyer Assessment Upper Limb (FMA-UL).

**Model**	**DF**	***F*-stat**	***p*-value**	**β**	***T*-stat**	***p*-value**
Model:	4,20	8.51	0.0004			
CST involvement				−0.22	−3.01	0.007
IPt				1.85	2.55	0.02
M1-M1 connectivity				23.65	2.52	0.02
PMd-PMv connectivity				−20.36	−2.03	0.06

Corticospinal tract (CST); trunk-based index of performance (IPt); left and right primary motor cortex (M1-M1) connectivity; right dorsal premotor and right ventral premotor cortex (PMd-PMv) connectivity; Degrees of freedom (DF); beta (β)-values.

We also performed two additional lasso regression analyses (see Supplemental Results). For the first analysis, we excluded data from any connectivity pair where one or more seed masks overlapped with the lesion mask; the pattern of findings are similar to those reported above. For the second analysis, we used the FMA-UL arm sub-score as the dependent variable; the pattern of findings are similar to those reported above.

## 4 Discussion

We found that the amount of CST involvement, IPt, and M1-M1 connectivity explained approximately 49 % of the variance in the FMA-UL full score. The addition of the IPt significantly contributed to the amount of explained variance in the FMA-UL full and arm sub-scores (14 % and 18 %, respectively). In contrast, ipsilesional connectivity when measured as a composite metric was not a significant explanatory variable in the present study sample. However, we found some evidence that ipsilesional connectivity within dorsal and ventral premotor cortex may account for some variance in the FMA-UL score. Together our findings show that motor impairment, as assessed with the FMA-UL, is best explained by a combination of neurophysiological and kinematic measures.

A novel finding is that the IPt significantly explained an additional 14 % of the variance in the FMA-UL full score compared to a model that only included CST involvement. The reaching task measures proximal (i.e. arm) movements while the FMA-UL assesses arm, hand, and finger movements. When we analyzed the data using the FMA-UL arm sub-score, the IPt accounted for 18 % of its variance. As the overall interpretation of our work is not changed whether the FMA-UL full or arm sub-score is used, we will discuss the findings in the context of the FMA-UL full score. The amount of CST involvement explained the most variance (stepwise model 1, R^2^_adj_ = 27 %) in motor impairment. Individuals with a stroke lesion that overlapped more with the CST had greater motor impairment than those with smaller overlaps. This was not unexpected since the integrity of the CST is crucial for motor behaviour of the distal upper limb (Lang and Schieber, 2004) and relationships between CST involvement and impairment have been previously reported (Lin et al., 2019, Quinlan et al., 2015, Lam et al., 2018). When both CST involvement and IPt explanatory variables were combined together, they accounted for 41 % of the variance in the FMA-UL score. The FMA-UL is a clinical assessment that evaluates impairment at the Body Structure/Function level of the International Classification of Functioning (Gladstone et al., 2002). It assesses whether movements are performed in or out of synergy, using an ordinal Likert-based scale that has low sensitivity to dissociate whether movements were performed with or without motor compensations. In contrast, the IPt is a continuous measure that assays the endpoint speed and accuracy of a functional reaching movement, while accounting for trunk compensation (Piscitelli et al., 2023). We previously showed it has a standard error of measurement of 14 % and minimal detectable change of 39 % (Piscitelli et al., 2023); however, studies are still required to determine its clinical relevance in the context of the minimal clinically important difference. While the IPt shares similar constructs with the FMA-UL (Piscitelli et al., 2023), it provides additional information about motor impairment (i.e. quantification of the degree of compensation) in the context of skilled motor performance. Our prior work showed that the IPt has a low-to-moderate relationship with the FMA-UL (R^2^ ranged from 0.236 to 0.428) (Piscitelli et al., 2023). ROC (receiver operating characteristic) analyses also suggested that the IPt distinguishes between levels of sensorimotor impairment severity based on the FMA-UL score (mild: ≥50/66 and moderate-to-severe:≤49/66) (Piscitelli et al., 2023). Similarly, the amount of CST involvement, quantified by the weighted lesion-load approach (Feng et al., 2015), or functionally by the motor evoked potential (Stinear et al., 2017), also discriminated between individuals with good versus poor recovery. Taken together, our findings suggest that the FMA-UL score reflects both structural information about the integrity of descending white matter pathways, and behavioural information about skilled performance. This suggests that both CST involvement and IPt are relevant biomarkers since they contribute to motor impairment.

It is worth pointing out that our prior work found CST involvement and M1-M1 connectivity to explain 50 % of the variance in motor impairment (Lam et al., 2018). Yet, in the present study, CST involvement, M1-M1 connectivity and the IPt explained a similar amount of variance (49 %). In our prior work, the IPt had not yet been developed and motor impairment was assessed with the Chedoke McMaster Stroke Assessment for the Arm and Hand (the FMA-UL was not collected). This highlights a challenge in current literature whereby different assessments are used, as well as approaches to evaluate CST involvement (Schuch et al., 2022) and methodologies to extract metrics associated with rsfMRI connectivity. Moreover, different imaging modalities (e.g. diffusion MRI, NODDI, etc.) and types of connectivity (e.g. task-based or network connectivity, etc.) can also be applied. Thus, the harmonization of protocols and analysis approaches is important to facilitate the comparison of findings across studies.

Prior rsfMRI studies showed that M1-M1 connectivity correlated with motor impairment and/or activity (Lam et al., 2018, Carter et al., 2010), suggesting that it may be a biomarker of improvements in motor behaviour (Boyd et al., 2017). In our study, M1-M1 connectivity explained an additional 8 % of the variance in the FMA-UL score as compared to a model that included both CST involvement and the IPt. Our findings support prior work showing that CST white matter integrity better predicted motor outcome than rsfMRI M1-M1 connectivity (Lin et al., 2018). This could in part be because interhemispheric rsfMRI M1-M1 connectivity reflects task-independent non-specific post-stroke motor reorganization (Paul et al., 2021) and therefore, explains recovery to a lesser extent. It is speculated that descending pathway integrity is more important to track changes in motor behaviour than grey matter cortical-cortical activity/connectivity (Branscheidt et al., 2022). Support for this notion comes from longitudinal studies that show no relationship between rsfMRI interhemispheric M1-M1 connectivity measures and changes in motor behaviour (Branscheidt et al., 2022, Nijboer et al., 2017). M1-M1 connectivity did not appear to change over time, despite changes in motor behaviour over the same period. However, other studies have shown rsfMRI M1-M1 connectivity to change over time post-stroke. In these studies, rsfMRI M1-M1 connectivity was initially reduced in the acute stage post-stroke, but in the late subacute to chronic stage post-stroke, was comparable to control participants (Park et al., 2011), especially in well recovered individuals (Golestani et al., 2013). While Branscheidt et al (2022) could not replicate (Branscheidt et al., 2022) these findings (Park et al., 2011, Golestani et al., 2013), it could also be that the degree to which cortical-cortical connectivity is implicated may depend on stroke severity. For example, Schulz et al (2017) showed that in individuals with greater disruption to the CST, white matter integrity between primary motor and ventral premotor cortex significantly explained variance in motor outcome (Schulz et al., 2017).

We also examined the role of ipsilesional rsfMRI connectivity between motor regions. The composite measure of ipsilesional connectivity did not significantly explain the variance in the FMA-UL score, in contrast to some evidence that specific seed-to-seed connectivity between dorsal and ventral premotor cortex did. On the one hand, prior work showed interhemispheric, but not ipsilesional, rsfMRI connectivity, correlated with post-stroke motor performance (Urbin et al., 2014). Longitudinally, rsfMRI ipsilesional connectivity also did not change over time (Nijboer et al., 2017). On the other hand, data from more complex network connectivity modeling and/or task-based dynamic causal modeling approaches suggest a role of ipsilesional regions. Studies using these methodologies have reported that ipsilesional premotor and primary motor connectivity explained ∼27 % of variance in motor performance (Paul et al., 2021) and is related to post-stroke motor impairment (Rehme et al., 2015). For example, excitatory coupling between ipsilesional premotor to M1 regions facilitated M1 activity related to paretic hand movements (Rehme et al., 2011). Further, enhanced ipsilesional connectivity may be more strongly associated with motor outcomes in people with severe impairment (Lee et al., 2017), potentially to support compensatory mechanisms (Paul et al., 2021). The role of dorsal and ventral premotor cortex in post-stroke recovery may be especially important. These regions are involved in reaching and grasping behaviours and when spared post-stroke, may be involved in motor recovery (Kantak et al., 2011).

Limitations of our study include the following. First, our observations are based on people living with late sub-acute to chronic stroke. Thus, the relationships described between neuroimaging and kinematic metrics with the FMA-UL outcome may not apply to other stages post-stroke. Our findings are still important as many people in these stages seek rehabilitation and about 65 % of stroke survivors present UL deficits despite rehabilitation interventions (Mayo et al., 2002). Recent evidence suggests that with intense rehabilitation, clinically meaningful significant gains can still be achieved in this population (Daly et al., 2019, McCabe et al., 2015, Ward et al., 2019). Importantly, these improvements may also take place at the impairment level (Daly et al., 2019, Ward et al., 2019). Identifying factors that explain a person’s motor impairment may help researchers design personalized interventions and clinicians tailor therapies that may be better focused on motor recovery/restitution goals. Second, our study population comprise predominantly of people with mild-to-moderate motor impairment (Woytowicz et al., 2017). We did not perform sub-group analyses given the small sample size combined with the absence of a principled approach in stratifying participants into meaningful categories. This would be important to do for future research as recent findings suggest that different subgroups of individuals with chronic stroke may show different patterns of rsfMRI connectivity (Rizor et al., 2024). This could explain why our measures of connectivity accounted for a smaller proportion of (or no) variance in the FMA-UL. Similarly, it could be relevant to understand whether the variance explained by the IPt differs depending on subgroups of participants. Third, our sample included more males (n = 22) than females (n = 3). We included all individuals who met study criteria and agreed to participate. This sex-based disparity in stroke research participation is not unique to our study (Carcel and Reeves, 2021). Future research should specifically target recruitment of females, as well as address other inequities. Fourth, our observations are cross-sectional with a relatively small sample. Longitudinal studies with large data sets are required to test whether these biomarkers, especially the IPt, predict outcomes from interventions. We have identified candidate biomarkers that can be further tested in such studies.

Fifth and lastly, the IPt addresses only one type of trunk compensation during reaching, however, there are other forms of compensation. These include altered movement patterns such as excessive shoulder abduction, trunk rotation, and lateral movements, etc. (Merdler et al., 2013, Levin et al., 2016). Thus, other kinematic measures may be identified that could influence the model. However, a challenge is that it may be difficult to identify a specific joint rotation or kinematic metric that can explain the most variance in motor impairment and that is sensitive to detecting change at all impairment levels for a given repertoire of upper limb movement. This is because movement patterns reflect a certain degree of variability for task specific actions due to the kinematic redundancy of the system (Bernstein, 1967, Latash, 2012).

## 5 Conclusion

The IPt is a kinematic biomarker of post-stroke reaching motor skill, and that, combined with the amount of CST involvement, accounts for ∼41 % of the variance in the FMA-UL score. These behavioural and structural biomarkers may be more relevant than resting state fMRI derived connectivity metrics that explain less (∼8% interhemispheric M1-M1 connectivity) or no (ipsilesional motor connectivity) variance in motor impairment measured by clinical scales.

## CRediT authorship contribution statement

**Joyce L. Chen:** Writing – review & editing, Writing – original draft, Validation, Supervision, Resources, Project administration, Methodology, Funding acquisition, Conceptualization. **Timothy K. Lam:** Writing – review & editing, Writing – original draft, Visualization, Validation, Software, Methodology, Investigation, Formal analysis, Data curation. **Melanie C. Baniña:** Writing – review & editing, Software, Investigation, Formal analysis, Data curation. **Daniele Piscitelli:** Writing – review & editing, Visualization, Validation, Software, Methodology, Formal analysis, Data curation, Conceptualization. **Mindy F. Levin:** Writing – review & editing, Supervision, Resources, Project administration, Methodology, Funding acquisition, Conceptualization.

## Funding

This work was funded by a Grant-in-Aid from the Heart and Stroke Foundation, Canada (G-16-00012613) to JLC and MFL.

## Declaration of Competing Interest

The authors declare that they have no known competing financial interests or personal relationships that could have appeared to influence the work reported in this paper.

## Data Availability

Data will be made available on request.
