# Supplementary material for: Neuroimaging and kinematic biomarkers of post-stroke upper limb motor impairment
Source: Neuroimage Clin. 2025 Jul 28;48:103854. doi: 10.1016/j.nicl.2025.103854 (PMC12356466; doi:10.1016/j.nicl.2025.103854)
Supplement: Supplementary Data 1 [file mmc1.docx]

**Supplementary Materials**

**Supplementary Methods**

***Resting state functional magnetic resonance imaging analyses – regressors of no interest***

Time series for six head motion parameters were obtained from MCFLIRT. For the CSF and WM, the T1-weighted image was segmented into grey matter, WM, and CSF using FMRIB’s Automated Segmentation Tool (FAST). This enabled us to produce binary CSF and WM masks, which we eroded twice to ensure no grey matter voxels were included as part of these masks. The CSF and WM masks were then registered to functional space using FMRIB’s Linear Image Registration Tool (FLIRT). Mean time series for the CSF and WM were extracted from voxels within the CSF and WM masks, respectively, from the preprocessed resting state fMRI data. A second FEAT analysis was performed whereby the time series of the six head motion parameters, CSF, and WM were inputted as regressors of no interest in the General Linear Model (GLM).

***Delineation of seed regions of interest for connectivity analyses***

To derive the seed coordinates for each region of interest, we first identified all studies from three meta-analyses (Mayka et al. 2006; Hardwick et al. 2018; Papitto et al. 2020) that utilized an arm/elbow fMRI or positron emission tomography (PET) paradigm. For studies that reported multiple coordinates in a motor region, such as two peak coordinates in the SMA for a reaching paradigm or two peak coordinates in the SMA for separate arm and elbow tasks, we computed the average coordinates from the given study. This enabled an equal contribution from each study to derive the average peak coordinates of a motor region despite different arm/elbow paradigms used across the selected studies. The process was done for each region of interest, M1, SMA, PMd, and PMv. The majority of arm/elbow studies identified in the three meta-analyses reported peak motor coordinates in the left hemisphere as the experimental paradigms were limited to the right arm/elbow. Therefore, we first computed the average coordinate in the left hemisphere for each motor region of interest using the peak coordinates reported in the arm/elbow studies. We then flipped the average coordinate for each motor region of interest along the mid-sagittal plane such that all coordinates were displayed on the right (ipsilesional) hemisphere.

**Supplementary Results**

***Stepwise and lasso regression – accounting for lesion-seed overlap***

In our sample, five participants have lesions that overlap with one or more seed masks. Three of these five participants have a lesion that overlaps with two or more seed masks. This latter point may be of concern as correlations between two seed masks that both overlap with the lesion may be spurious (Yourganov et al. 2018).

| **Participant** | **% right M1 seed overlap with lesion** | **% right SMA seed overlap with lesion** | **% right PMd seed overlap with lesion** | **% right PMv seed overlap with lesion** |
| --- | --- | --- | --- | --- |
| S01 | 0 | 0 | 0 | 0 |
| S02 | 0 | 0 | 0 | 0 |
| S03 | 0 | 0 | 0 | 0 |
| S04 | 0 | 0 | 0 | 0 |
| S05 | 0 | 0 | 0 | 0 |
| S06 | 0 | 0 | 0 | 0 |
| S07 | 0 | 0 | 0 | 0 |
| S08 | 0 | 0 | 0 | 0 |
| S09 | 0 | 0 | 0 | 0 |
| S10 | 0 | 0 | 0 | 0 |
| S11 | 0 | 0 | 0 | 0 |
| S12 | 0 | 0 | 0 | 0 |
| S13 | 0 | 0 | 0 | 77.24 |
| S14 | 36.58 | 0 | 10.57 | 84.55 |
| S15 | 0 | 0 | 0 | 0 |
| S16 | 0 | 0 | 0 | 0 |
| S17 | 0 | 0 | 0 | 0 |
| S18 | 0 | 0 | 0 | 0 |
| S19 | 0 | 0 | 0 | 0 |
| S20 | 0 | 43.09 | 8.13 | 0 |
| S21 | 0.81 | 0 | 11.38 | 35.77 |
| S22 | 0 | 0 | 0 | 8.13 |
| S23 | 0 | 0 | 0 | 0 |
| S24 | 0 | 0 | 0 | 0 |
| S25 | 0 | 0 | 0 | 0 |

**Stepwise regression model**

We performed an analysis where we excluded data from any connectivity pair where one or more seed masks overlapped with the lesion mask. For example, in s13, the RPMv seed mask overlaps with the lesion mask. Therefore, for this participant, we excluded connectivity measures of RM1-RPMv, RSMA-RPMv, and RPMd-RPMv; only RM1-RSMA and RM1-RPMd connectivity values were calculated and used to inform Ipsilesional Connectivity. For s14 and s21, this meant that no connectivity measures were derived since any combination of seed masks was confounded with a lesion-seed mask overlap. In these two participants (s14, s21), we adopted a conservative approach by inputting ‘zero’ for their ipsilesional connectivity values. We performed the stepwise regression model and the findings do not change from those originally reported; they are identical. This is because the relationship between ipsilesional connectivity and the FMA-UL remained non-significant and therefore does not enter into the model.

We also performed the analysis with data from s14 and s21 excluded. M1-M1 connectivity is no longer significant but the IPt finding remains significant.

| **Model** | **R^2^** | **Adjusted R^2^** | ***p*-value** | **β**  **[95% CI]** | ***p*-value** | **ΔR^2^_adj_** | ***p*-value** |
| --- | --- | --- | --- | --- | --- | --- | --- |
| Model 1:  CST involvement | 0.30 | 0.27 | 0.006 | −0.55 [-0.48, -0.09] | 0.006 | - | - |
|  |  |  |  |  |  |  |  |
| Model 2:  CST involvement  IPt | 0.46 | 0.41 | 0.002 | −0.48 [-0.43, -0.07]  0.40 [0.26,3.83] | 0.009  0.027 | 0.14 | 0.03 |
|  |  |  |  |  |  |  |  |

**Lasso regression model**

We performed the Lasso regression with the updated ipsilesional connectivity values (both average across all pairs, and individual pairs of connectivity) and the pattern of findings are similar to those reported in the main text. The selection process resulted in 4 variables being retained: CST involvement, IPt, M1-M1 connectivity, and PMd-PMv connectivity.

| **Model** | **DF** | ***F*-stat** | ***p*-value** | **β** | ***T*-stat** | ***p*-value** |
| --- | --- | --- | --- | --- | --- | --- |
| Model:  CST involvement  IPt  M1-M1 connectivity  PMd-PMv connectivity | 4,20 | 8.48 | 0.0004 | −0.23  1.86  22.55  −16.99 | −3.27  2.56  2.41  −2.01 | 0.004  0.02  0.03  0.06 |

***Stepwise and lasso regression - explained variance in the FMA-UL arm sub-score***

The IPt captures a goal-directed reaching movement without necessarily involving the hand and fingers. Therefore, we performed analyses using the FMA-UL arm sub-score as the dependent measure. We defined the arm sub-score based on the original assessment by Fugl-Meyer from the University of Gothenburg: <https://www.gu.se/en/neuroscience-physiology/fugl-meyer-assessment>. Therefore, the total score is out of 36 points (and excludes wrist, hand, and coordination/speed components of the assessment).

For each of the stepwise and lasso regressions, we performed two versions of the analyses, one with the original ipsilesional connectivity values, and one that accounts for the lesion - seed mask overlap. Both approaches yield the same findings. Below we report analyses conducted with the lesion – seed mask overlap accounted for.

The pattern of findings with the stepwise regression are slightly modified. The model that is significant only includes the IPt and CST involvement, which together account for approximately 30% of the variance in the FMA-UL arm sub-score. M1-M1 connectivity no longer significantly explains variance in the FMA arm sub-score.

**Stepwise regression model**

| **Model** | **R^2^** | **Adjusted R^2^** | ***p*-value** | **β**  **[95% CI]** | ***p*-value** | **ΔR^2^_adj_** | ***p*-value** |
| --- | --- | --- | --- | --- | --- | --- | --- |
| Model 1:  IPt | 0.22 | 0.18 | 0.02 | 0.46 [0.20, 2.04] | 0.02 | - | - |
|  |  |  |  |  |  |  |  |
| Model 2:  IPt  CST involvement | 0.36 | 0.30 | 0.007 | 0.39 [0.08, 1.82]  −0.39 [−0.18, −0.008] | 0.03  0.03 | 0.12 | 0.03 |

The pattern of findings with the lasso regression are similar to those presented with the FMA-UL full score.

**Lasso regression model**

| **Model** | **DF** | ***F*-stat** | ***p*-value** | **β** | ***T*-stat** | ***p*-value** |
| --- | --- | --- | --- | --- | --- | --- |
| Model:  CST involvement  IPt  M1-M1 connectivity  PMd-PMv connectivity | 4,20 | 6.65 | 0.001 | −0.09  0.93  8.54  −11.46 | −2.54  2.52  1.80  −2.67 | 0.02  0.02  0.09  0.01 |

**Supplementary Tables**

**Supplementary Table S1**: Peak coordinates from studies that used an arm/elbow paradigm to derive the primary motor cortex (M1) seed

| **First Author (Year)** | **Paradigm** | **Imaging Modality** | **X** | **Y** | **Z** |
| --- | --- | --- | --- | --- | --- |
| Alkadhi (2002) | Elbow flexion/extension | fMRI | −29 | −25 | 61 |
| Desmurget (2001)* | Pointing task | PET | −35 | −26.25 | 56.5 |
| Ghilardi (2000)* | Reaching task | PET | −35 | −19 | 63 |
| Grafton (1993)* | Visuomotor tracking | PET | −22 | −23 | 60 |
| Kawashima (1998)* | Arm movement | PET | −24 | −25.5 | 62.5 |
| Lacquaniti (1997)* | Pointing task | PET | −32 | −24 | 52 |
| Nyberg (2001)* | Upper limb movement | PET | −40.25 | −13.75 | 42 |
| Osborne (2015) | Arm movements | fMRI | −30 | −25 | 58 |
| Tracy (2001) | Bimanual arm rotation | fMRI | −46 | −16 | 30 |
| Turner (1998)* | Tracking task | PET | −36 | −28.3 | 54.7 |
| Vingerhoets (2011) | Pantomiming tools | fMRI | −33 | −22 | 57 |
| Weiss (2003) | Reaching task | PET | −50 | −30 | 58 |
|  |  |  |  |  |  |
| **Average Coordinate** |  |  | −34 | −23 | 54 |

*Asterisk represents studies that listed multiple peak coordinates for the M1. For these studies, the coordinate presented in this table is the average of the peak coordinates.

Note: Majority of studies reported peak coordinates in the left hemisphere since these studies used a paradigm that involved the right arm/elbow. To derive the M1 seed for the right (ipsilesional) hemisphere, we flipped the average coordinate listed in this table along the mid-sagittal plane.

**Supplementary Table S2**: Peak coordinates from studies that used an arm/elbow paradigm to derive the supplementary motor area (SMA) seed

| **First Author (Year)** | **Paradigm** | **Imaging Modality** | **X** | **Y** | **Z** |
| --- | --- | --- | --- | --- | --- |
| Grafton (1993)* | Visuomotor tracking | PET | −3 | −11 | 50.5 |
| Inoue (2000) | Reaching task | PET | −12 | −22 | 54 |
| Johnson (2002) | Underhand and overhand grip | fMRI | −18 | −6 | 53 |
| Kawashima (1998) | Arm movement | PET | −5 | −18 | 63 |
| Lacquaniti (1997)* | Pointing task | PET | −9 | −11 | 48 |
| Nyberg (2001) | Upper limb movement | PET | −2 | −7 | 46 |
| Tracy (2001) | Bimanual arm rotation | fMRI | −2 | −6 | 58 |
| Turner (1998) | Tracking task | PET | −13 | −18 | 55 |
| Vingerhoets (2011)* | Pantomiming tools | fMRI | −4.67 | 0 | 55 |
|  |  |  |  |  |  |
| **Average Coordinate** |  |  | −8 | −11 | 54 |

*Asterisk represents studies that listed multiple peak coordinates for the SMA. For these studies, the coordinate presented in this table is the average of the peak coordinates.

Note: Majority of studies reported peak coordinates in the left hemisphere since these studies used a paradigm that involved the right arm/elbow. To derive the SMA seed for the right (ipsilesional) hemisphere, we flipped the average coordinate listed in this table along the mid-sagittal plane.

**Supplementary Table S3**: Peak coordinates from studies that used an arm/elbow paradigm to derive the dorsal premotor cortex (PMd) seed

| **First Author (Year)** | **Paradigm** | **Imaging Modality** | **X** | **Y** | **Z** |
| --- | --- | --- | --- | --- | --- |
| Desmurget (2001)* | Pointing task | PET | −48 | −24 | 55.5 |
| Ghilardi (2000) | Reaching task | PET | −19.3 | −8 | 64.7 |
| Inoue (2000)* | Reaching task | PET | −34 | 5 | 57 |
| Kawashima (1998)* | Arm movement | PET | −21 | −11 | 63.5 |
| Lacquaniti (1997) | Pointing task | PET | −36 | 0 | 12 |
| Nyberg (2001)* | Upper limb movement | PET | −26 | −5 | 53.25 |
| Tracy (2001) | Bimanual arm rotation | fMRI | −34 | −14 | 66 |
| Turner (1998) | Tracking task | PET | −31 | −16 | 64 |
| Vingerhoets (2011)* | Pantomiming tools | fMRI | −23.5 | −16.5 | 59.5 |
| Weiss (2003)* | Reaching task | PET | −25 | −1 | 57 |
|  |  |  |  |  |  |
| **Average Coordinate** |  |  | −30 | −9 | 55 |

*Asterisk represents studies that listed multiple peak coordinates for the PMd. For these studies, the coordinate presented in this table is the average of the peak coordinates.

Note: Majority of studies reported peak coordinates in the left hemisphere since these studies used a paradigm that involved the right arm/elbow. To derive the PMd seed for the right (ipsilesional) hemisphere, we flipped the average coordinate listed in this table along the mid-sagittal plane.

**Supplementary Table S4**: Peak coordinates from studies that used an arm/elbow paradigm to derive the ventral premotor cortex (PMv) seed

| **First Author (Year)** | **Paradigm** | **Imaging Modality** | **X** | **Y** | **Z** |
| --- | --- | --- | --- | --- | --- |
| Kawashima (1996) | Arm movement | PET | −56 | 7 | 23 |
| Lacquaniti (1997) | Pointing task | PET | −54 | 2 | 16 |
| Nyberg (2001)* | Upper limb movement | PET | −49.5 | 5.5 | 12 |
| Turner (1998) | Tracking task | PET | −45 | −1 | 51 |
|  |  |  |  |  |  |
| **Average Coordinate** |  |  | −51 | 3 | 26 |

*Asterisk represents studies that listed multiple peak coordinates for the PMv. For these studies, the coordinate presented in this table is the average of the peak coordinates.

Note: Majority of studies reported peak coordinates in the left hemisphere since these studies used a paradigm that involved the right arm/elbow. To derive the PMv seed for the right (ipsilesional) hemisphere, we flipped the average coordinate listed in this table along the mid-sagittal plane.

**Supplementary Table S5**: Collinearity assessment of the predictor variables in the stepwise regression models

| **Model** | **VIF** |
| --- | --- |
| Model 1:  CST involvement | 1.000 |
|  |  |
| Model 2:  CST involvement  IPt | 1.034  1.034 |
|  |  |
| Model 3:  CST involvement  IPt  M1-M1 connectivity | 1.046  1.078  1.066 |

Abbreviations: VIF: variance inflation factor. VIF values are listed for the predictor variables included in the interim and final models of the stepwise regression analysis (see Table 2 of the main manuscript). VIF values of 10 or higher indicate high multicollinearity.

**Supplementary Table S6**: Spearman’s Rho (ρ) correlation between biomarkers and demographic variables

|  | **Age** | **Sex** | **Time since stroke** | **Lesioned hemisphere** | **Dominant hand affected** |
| --- | --- | --- | --- | --- | --- |
| **IPt** | ρ = −0.08  p = 0.70 | ρ = 0.22  p = 0.29 | ρ = −0.05  p = 0.81 | ρ = 0.17  p = 0.43 | ρ = −0.02  p = 0.91 |
| **CST involvement** | ρ = −0.28  p = 0.17 | ρ = 0.03  p = 0.87 | ρ = 0.11  p = 0.61 | ρ = 0.46  p = 0.02* | ρ = 0.42  p = 0.04* |
| **M1-M1 connectivity** | ρ = 0.10  p = 0.63 | ρ = 0.05  p = 0.81 | ρ = 0.08  p = 0.70 | ρ = −0.39  p = 0.06 | ρ = −0.50  p = 0.01* |
| **Ipsilesional connectivity** | ρ = 0.13  p = 0.54 | ρ = −0.15  p = 0.46 | ρ = −0.29  p = 0.16 | ρ = 0.14  p = 0.49 | ρ = −0.04  p = 0.83 |
| **FMA-UL** | ρ = 0.25  p = 0.22 | ρ = 0.32  p = 0.11 | ρ = −0.31  p = 0.14 | ρ = −0.28  p = 0.18 | ρ = −0.25  p = 0.23 |

**Asterisk represents a significant correlation at p < 0.05.*

**Supplementary Table S7**: Participant demographics

| **Participant** | **Age** | **Sex** | **Time since stroke (months)** | **Lesioned hemisphere** | **Lesion volume (cc)** | **Dominant hand affected** | **FMA-UL** |
| --- | --- | --- | --- | --- | --- | --- | --- |
| **s01** | 66 | M | 8.1 | L | 0.82 | Y | 56 |
| **s02** | 54 | M | 57.0 | R | 10.96 | Y | 41 |
| **s03** | 61 | M | 25.8 | R | 50.16 | N | 50 |
| **s04** | 48 | M | 12 | R | 4.66 | N | 34 |
| **s05** | 64 | F | 4.2 | L | 16.94 | N | 55 |
| **s06** | 57 | M | 8.4 | L | 5.82 | Y | 50 |
| **s07** | 77 | M | 43.2 | L | 17.54 | N | 45 |
| **s08** | 68 | M | 31.6 | R | 3.68 | N | 48 |
| **s09** | 39 | M | 8.3 | L | 1.44 | Y | 57 |
| **s10** | 70 | M | 20 | R | 18.25 | N | 41 |
| **s11** | 74 | F | 11 | L | 14.47 | Y | 56 |
| **s12** | 66 | M | 10.2 | R | 23.42 | N | 21 |
| **s13** | 38 | M | 46.7 | R | 193.98 | N | 28 |
| **s14** | 86 | M | 8.6 | R | 53.98 | N | 35 |
| **s15** | 66 | M | 6.2 | R | 4.32 | N | 59 |
| **s16** | 60 | M | 4.8 | L | 2.28 | Y | 45 |
| **s17** | 65 | M | 4.5 | R | 3.26 | N | 44 |
| **s18** | 62 | M | 3.6 | R | 3.87 | N | 65 |
| **s19** | 50 | M | 6 | L | 9.30 | Y | 27 |
| **s20** | 65 | M | 17.4 | L | 39.27 | Y | 60 |
| **s21** | 58 | F | 10.4 | R | 81.14 | N | 56 |
| **s22** | 62 | M | 61.3 | L | 12.99 | N | 39 |
| **s23** | 38 | M | 9.1 | R | 22.40 | N | 13 |
| **s24** | 54 | M | 27.8 | L | 3.86 | Y | 43 |
| **s25** | 67 | M | 14.8 | L | 1.10 | Y | 50 |

Abbreviations: cc: cubic centimeters; FMA-UL: Fugl-Meyer Assessment Upper Limb score; M: male; F: female; L: left; R: right; Y: yes; N: no. The time since stroke for participant s22 was one month greater than the cut-off criteria for stroke onset of < 5 years.

**Supplementary Table S8**: Neuroimaging and kinematic biomarker values

| **Participant** | **IPt** | **CST involvement** | **M1-M1 connectivity** | **Ipsilesional connectivity** |
| --- | --- | --- | --- | --- |
| **s01** | 4.31 | 13.75 | 0.28 | 0.25 |
| **s02** | 6.22 | 94.83 | 0.76 | 0.61 |
| **s03** | 5.65 | 57.58 | 0.47 | 0.18 |
| **s04** | 6.61 | 58.18 | 0.36 | 0.45 |
| **s05** | 5.09 | 55.71 | 0.60 | 0.46 |
| **s06** | 2.08 | 73.58 | 0.77 | 0.44 |
| **s07** | 4.54 | 54.55 | 0.38 | 0.30 |
| **s08** | 4.56 | 42.31 | 0.75 | 0.50 |
| **s09** | 7.31 | 21.43 | 0.47 | 0.32 |
| **s10** | 1.72 | 53.03 | 0.33 | 0.31 |
| **s11** | 5.20 | 55.77 | 0.69 | 0.30 |
| **s12** | 5.18 | 77.14 | 0.02 | 0.35 |
| **s13** | 2.34 | 87.14 | 0.26 | 0.25 |
| **s14** | 2.35 | 100.00 | 0.43 | 0.35 |
| **s15** | 5.63 | 63.46 | 0.37 | 0.41 |
| **s16** | 2.23 | 29.09 | 0.39 | 0.19 |
| **s17** | 1.62 | 61.82 | 0.29 | 0.54 |
| **s18** | 12.86 | 62.26 | 0.61 | 0.51 |
| **s19** | 3.43 | 78.79 | 0.62 | 0.51 |
| **s20** | 7.86 | 44.44 | 0.61 | 0.36 |
| **s21** | 5.74 | 84.75 | 0.25 | 0.13 |
| **s22** | 1.68 | 100.00 | 0.45 | 0.21 |
| **s23** | 3.52 | 98.08 | 0.19 | 0.25 |
| **s24** | 2.28 | 53.45 | 0.39 | 0.08 |
| **s25** | 4.00 | 20.75 | 0.66 | 0.48 |

Abbreviations: IPt: trunk-based index of performance; CST: corticospinal tract; M1-M1: left and right primary motor cortex.

**References**

Alkadhi H, Crelier GR, Hotz Boendermaker S, Golay X, Hepp-Reymond MC, Kollias SS. Reproducibility of primary motor cortex somatotopy under controlled conditions. *AJNR Am J Neuroradiol*. 2002;23(9):1524-1532.

Desmurget M, Gréa H, Grethe JS, Prablanc C, Alexander GE, Grafton ST. Functional anatomy of nonvisual feedback loops during reaching: a positron emission tomography study. *J Neurosci*. 2001;21(8):2919-2928.

Ghilardi M, Ghez C, Dhawan V, et al. Patterns of regional brain activation associated with different forms of motor learning. *Brain Res*. 2000;871(1):127-145.

Grafton ST, Woods RP, Mazziotta JC. Within-arm somatotopy in human motor areas determined by positron emission tomography imaging of cerebral blood flow. *Exp Brain Res*. 1993;95(1):172-176.

Hardwick RM, Caspers S, Eickhoff SB, Swinnen SP. Neural correlates of action: Comparing meta-analyses of imagery, observation, and execution. *Neurosci Biobehav Rev*. 2018;94:31-44.

Inoue K, Kawashima R, Satoh K, et al. A PET study of visuomotor learning under optical rotation. *Neuroimage*. 2000;11(5 Pt 1):505-516.

Johnson SH, Rotte M, Grafton ST, Hinrichs H, Gazzaniga MS, Heinze HJ. Selective activation of a parietofrontal circuit during implicitly imagined prehension. *Neuroimage*. 2002;17(4):1693-1704.

Kawashima R, Itoh H, Ono S, et al. Changes in regional cerebral blood flow during self-paced arm and finger movements. A PET study. *Brain Res*. 1996;716(1-2):141-148.

Lacquaniti F, Perani D, Guigon E, et al. Visuomotor transformations for reaching to memorized targets: a PET study. *Neuroimage*. 1997;5(2):129-146.

Mayka MA, Corcos DM, Leurgans SE, Vaillancourt DE. Three-dimensional locations and boundaries of motor and premotor cortices as defined by functional brain imaging: a meta-analysis. *Neuroimage*. 2006;31(4):1453-1474.

Nyberg L, Petersson KM, Nilsson LG, Sandblom J, Aberg C, Ingvar M. Reactivation of motor brain areas during explicit memory for actions. *Neuroimage*. 2001;14(2):521-528.

Osborne NR, Owen AM, Fernández-Espejo D. The dissociation between command following and communication in disorders of consciousness: an fMRI study in healthy subjects. *Front Hum Neurosci*. 2015;9:493.

Papitto G, Friederici AD, Zaccarella E. The topographical organization of motor processing: An ALE meta-analysis on six action domains and the relevance of Broca's region. *Neuroimage*. 2020;206:116321.

Tracy JI, Faro SS, Mohammed FB, Pinus AB, Madi SM, Laskas JW. Cerebellar mediation of the complexity of bimanual compared to unimanual movements. *Neurology*. 2001;57(10):1862-1869.

Turner RS, Grafton ST, Votaw JR, Delong MR, Hoffman JM. Motor subcircuits mediating the control of movement velocity: a PET study. *J Neurophysiol*. 1998;80(4):2162-2176.

Vingerhoets G, Vandekerckhove E, Honoré P, Vandemaele P, Achten E. Neural correlates of pantomiming familiar and unfamiliar tools: action semantics versus mechanical problem solving? *Hum Brain Mapp*. 2011;32(6):905-918.

Weiss PH, Marshall JC, Zilles K, Fink GR. Are action and perception in near and far space additive or interactive factors? *Neuroimage*. 2003;18(4):837-846.

Yourganov G, Fridriksson J, Stark B, Rorden C. Removal of artifacts from resting-state fMRI data in stroke. *NeuroImage: Clinical*. 2018;17:297-305.
